# Global patterns and trends in ovarian cancer incidence: age, period and birth cohort analysis

**DOI:** 10.1186/s12885-019-6139-6

**Published:** 2019-10-22

**Authors:** Yanting Zhang, Ganfeng Luo, Mengjie Li, Pi Guo, Yuejiao Xiao, Huanlin Ji, Yuantao Hao

**Affiliations:** 1 Department of Medical Statistics and Epidemiology, School of Public Health, Sun Yat-sen University, Guangzhou, 510080 China; 2 School of Public Health (Shenzhen), Sun Yat-sen University, Shenzhen, 518107 China; 3 Department of Public Health, Shantou University Medical College, Shantou, 515041 China; 4 Sun Yat-sen Global Health Institute, Sun Yat-sen University, Guangzhou, No.74 Zhongshan 2nd Rd, Guangzhou, 510000 China

**Keywords:** Ovarian cancer, Incidence, Global variations, Trends, Birth cohort

## Abstract

**Background:**

Ovarian cancer (OC) is the seventh most common malignancy worldwide and the most lethal gynaecological malignancy. We aimed to explore global geographical patterns and temporal trends from 1973 to 2015 for 41 countries in OC incidence and especially to analyse the birth cohort effect to gain further insight into the underlying causal factors of OC and identify countries with increasing risk of OC.

**Methods:**

OC data were drawn from the Cancer Incidence in Five Continents databases and online databases published by governments. The joinpoint regression model was applied to detect changes in OC trends. The age–period–cohort model was applied to explore age and birth cohort effects.

**Results:**

The age-standardized rate of OC incidence ranged from 3.0 to 11.4 per 100,000 women worldwide in 2012. The highest age-standardized rate was observed in Central and Eastern Europe, with 11.4 per 100,000 women in 2012. For the most recent 10-year period, the increasing trends were mainly observed in Central and South America, Asia and Central and Eastern Europe. The largest significant increase was observed in Brazil, with an average annual percentage change of 4.4%. For recent birth cohorts, cohort-specific increases in risk were pronounced in Estonia, Finland, Iceland, Lithuania, the United Kingdom, Germany, the Netherlands, Italy, Malta, Slovenia, Bulgaria, Russia, Australia, New Zealand, Brazil, Costa Rica, Ecuador, India, Japan, the Philippines and Thailand.

**Conclusions:**

Disparities in the incidence and risk of OC persist worldwide. The increased risk of birth cohort in OC incidence was observed for most countries in Asia, Central and Eastern Europe, and Central and South America. The reason for the increasing OC risk for recent birth cohorts in these countries should be investigated with further epidemiology studies.

## Background

Ovarian cancer (OC) is the seventh most common malignancy worldwide, with 238,719 newly diagnosed cases in 2012 [1]. The incidence of OC has appreciable geographic variation worldwide [1]. OC is more frequently diagnosed at an advanced stage, and its prognosis is poor, which makes this cancer the most lethal gynaecological malignancy [2]. Thus, understanding the aetiology of OC and identifying the causal factors and populations at high risk are essential for primary prevention.

To better understand the effect of lifestyle factors or reproductive patterns on OC incidence, we conducted an age–period–cohort analysis to explore the effect of birth cohort [3–6]. Birth cohort effects can reflect the long-established generational effect of causal factors in cancer incidence [3–6]. For example, a recent age–period–cohort analysis in Japan, the Republic of Korea and Singapore indicated that the increased risk of OC in younger birth cohorts was caused by changes in reproductive patterns and a shift towards a westernized lifestyle and dietary factors [4]. To date, no study has examined global trends in OC by an age–period–cohort analysis, which has been found to be more useful than a conventional cross-sectional analysis in evaluating trends [7].

We aimed to explore global geographical patterns of OC incidence and temporal trends from 1973 to 2015 for 41 countries. In particular, we aimed to analyse the birth cohort effect to examine the importance of changes in lifestyle or reproductive patterns, identify countries with increasing risk of OC and highlight trends that deserve closer attention by public health and cancer prevention specialists.

## Methods

OC incidence data were categorized according to the International Classification of Disease for Oncology (ICD-O), 3rd edition (C56). The data for the age-standardized rate (ASR) of OC incidence in 2012 for regions and 184 countries worldwide were drawn from the GLOBOCAN 2012 database [8], which formulated national estimates of cancer incidence from the best available data source (often based on data from Cancer Incidence in Five Continents (CI5)) and weighted averages of regional data in each country, with variable levels of accuracy depending on the extent and validity of locally available data [9]. We also extracted long-term data from the CI5, Volumes IV–X, the CI5plus database, and online databases published by governments. CI5 is the main source of high-quality global cancer incidence data for validity, completeness, and comparability [10]. Incidence data for the United States (USA) were extracted from the Surveillance, Epidemiology and End Results Program (SEER), which represents the most reliable data source of cancer incidence in the USA [11]. Although these databases, including GLOBOCAN, CI5 and SEER, have been used extensively in studies examining the global patterns and trends in the incidence of various cancers [12–14], there are some possible heterogeneities of these databases and different registries in our study. For example, GLOBOCAN includes simulated figures different from the active data collection of CI5 and SEER. The discrepancy between simulated figures of GLOBOCAN and the accurate data of CI5 and SEER is likely to be greater in countries with lower quality cancer registry data. The accuracy of data in these databases also varies from region to region and in different registries, especially for developing countries. Caution should be taken when interpreting the findings of developing countries.

To examine the trends in OC incidence, the inclusion requirement in our study was a continuous data of at least 15 years and containment in the last volume of the CI5 series (Volume X) to avoid statistical instability and ensure the quality of the data [6, 15]. Finally, 41 countries were selected. Of these countries, the incidence data for 26 countries were at the national level. For the remaining countries with two or more cancer registries, we pooled the cases and population data in all registries to cover the largest geographic area with an estimated national level [3].

We calculated a summary ASR using direct standardization with the world standard population [16]. To examine the geographic diversity in OC incidence, ASR by region and 184 countries in 2012 were plotted. To graphically present the trend in OC ASR, we performed locally weighted scatterplot smoothing (LOWESS) regression to fit smoothed lines [17]. To examine the changes in ASR, we performed a joinpoint regression model to calculate the annual percent change and the average annual percent change (AAPC) [17].

We conducted age-period-cohort analyses in all 41 countries. We subtracted the midpoints of 5-year age groups (20–24, 25–29, …, 80–84) from the corresponding 1-year calendar periods of diagnosis to obtain birth cohorts. Finally, we described the magnitude of the rates λ(a, p) as a function of age (a), period (p) and birth cohort (c) using a log-linear model, with Poisson distribution and with the log of the person-years at risk defined as an offset [3, 5, 6]:

$$ \left[\log \Big(\uplambda \left(\mathrm{a},\mathrm{p}\Big)\right)={\upalpha}_{\mathrm{a}}+{\upbeta}_{\mathrm{p}}+{\upgamma}_{\mathrm{c}}\right] $$

We applied a full age–period–cohort model to estimate birth cohort effects with incidence rate ratio (IRR) relative to the reference birth cohort. To overcome the non-identifiability problem of the linear dependence between three factors, we constrained the linear component of the period effect to have a zero slope, assuming that the linear changes in OC incidence resulted from cohort-related factors [3, 5, 6]. This statistical method has been widely applied in many published papers about global trends in the incidence of other cancers [3, 5, 6].

The global map was depicted by using ArcGIS (version 10.2). The figures were drawn by using Sigma Plot (version 12.5). Joinpoint regression models were performed by the Joinpoint Regression Program (version 4.3.1.0). The age–period–cohort model analyses and graphs were conducted using APCfit in Stata (version 13.0).

## Results

**Fig. 1:**
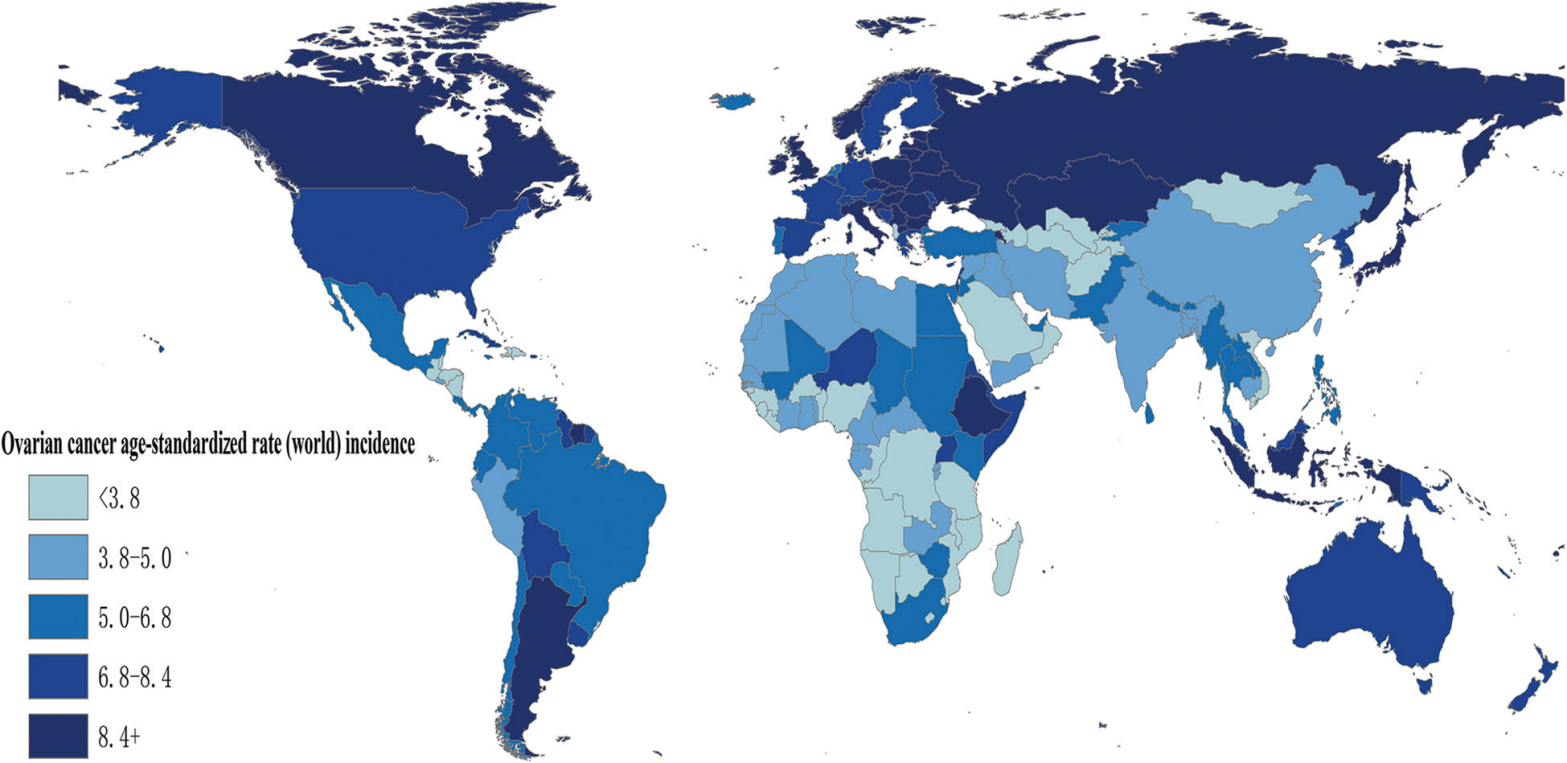
Estimated international variation in age-standardized (world) ovarian cancer incidence rates for all ages. National OC incidence estimates in 2012 for 184 countries were extracted from the GLOBOCAN 2012 database (http://globocan.iarc.fr). The map was depicted by ourselves using ArcGIS v10.2 software

**Fig. 2:**
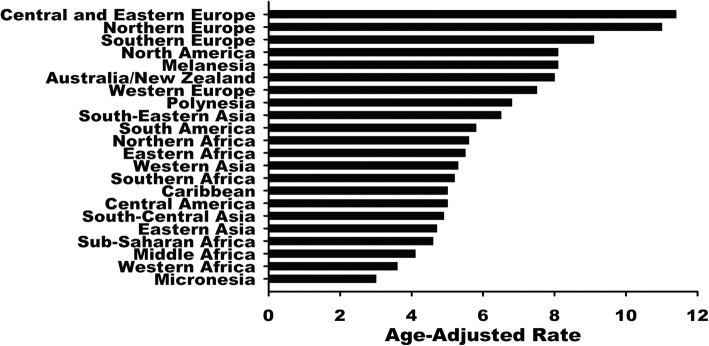
Estimated age-standardized (world) ovarian cancer incidence rates for all ages for all regions. Data were extracted from the GLOBOCAN 2012 database (http://globocan.iarc.fr)

There were an estimated 238,719 incident cases of OC and an ASR of 6.1 per 100,000 women worldwide in 2012 (Table 1). Approximately 9.2 per 100,000 women of ASR occurred in more developed regions and 5 per 100,000 women in less developed regions. The highest ASR was observed in Central and Eastern Europe, with 11.4 per 100,000 women, while the lowest ASR was observed in Micronesia, with 3.0 per 100,000 women (Fig. 1 and Fig. 2).

**Table 1:** Estimated number of ovarian cancer incident cases by region of the world in 2012

Area	Female Population (thousands)	Incident cases	ASR
World	3,496,728	238,719	6.1
By development level			
More developed regions	639,750	99,752	9.2
Less developed regions	2,856,978	138,967	5.0
By human development level			
Very high human development	583,579	84,723	8.5
High human development	529,972	45,263	7.3
Medium human development	1,732,608	87,421	4.7
Low human development	649,116	21,203	4.7
Africa	536,226	17,755	4.8
Sub-Saharan Africa	432,311	12,705	4.6
Eastern Africa	176,946	5907	5.5
Middle Africa	66,998	1561	4.1
Northern Africa	103,915	5050	5.6
Southern Africa	29,530	1417	5.2
Western Africa	158,836	3820	3.6
Central and South America and Caribbean	305,376	17,921	5.6
Caribbean	21,261	1205	5.0
Central America	81,200	3918	5.0
South America	202,913	12,798	5.8
North America	177,315	23,529	8.1
Asia	2,075,183	111,887	5.0
Eastern Asia	769,611	48,341	4.7
South-Eastern Asia	304,912	19,932	6.5
South-Central Asia	884,657	38,388	4.9
Western Asia	116,002	5226	5.3
Europe	383,793	65,584	9.9
Central and Eastern Europe	156,037	28,259	11.4
Northern Europe	50,931	10,023	11
Southern Europe	80,058	12,872	9.1
Western Europe	96,765	14,430	7.5
Oceania	18,833	2043	8.0
Australia/New Zealand	13,758	1718	7.6
Melanesia	4468	294	8.1
Micronesia	271	9	3.0
Polynesia	334	22	6.8

*ASR* age-standardized rate. Human Development Index (HDI) is a summary index of life expectancy, education period, and income per capita. The HDI was defined as low (< 0.534), medium (0.534–0.710), high (0.710–0.796) and very high (> 0.796)

**Fig. 3:**
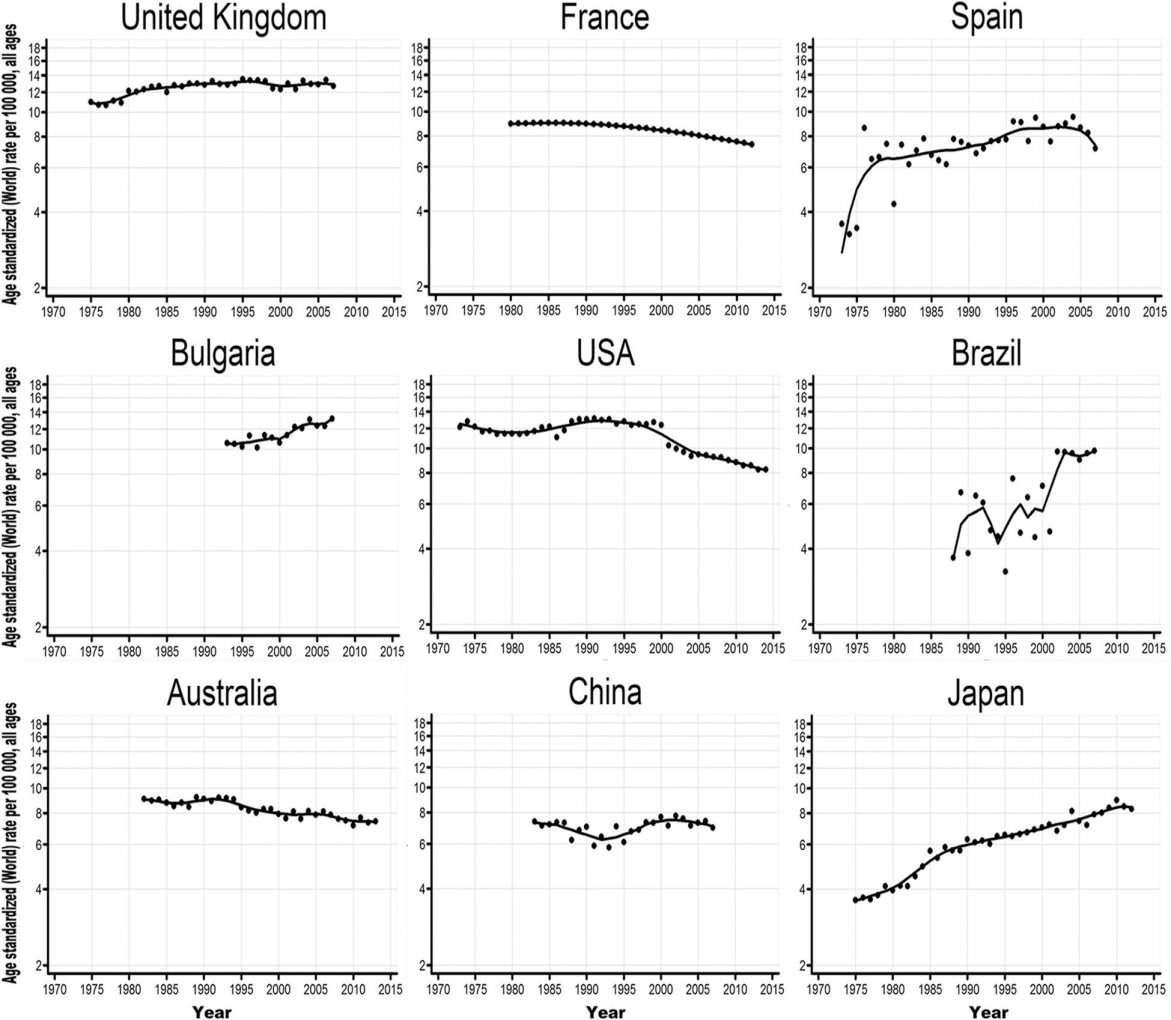
Temporal trends in age-standardized (world 1960 Segi population) ovarian cancer incidence rates per 100,000 women for nine selected countries from each region for all ages from 1973 to 2015

Table 2 shows temporal trends of ASR from 1973 onward as well as the AAPC values for the last 10-year period. The scatter plots with LOWESS regression curves are shown in Fig. 3, Additional file 1: Figure S1 and S2 (nine exemplary countries are shown in the text and the remaining countries are shown in supplemental figures to make the figure clearer). Over the entire study period, the ASR increased continually in Brazil, Colombia, Ecuador, Costa Rica, Germany, Estonia, Latvia, Lithuania, the United Kingdom (UK), Spain, Bulgaria, Poland, Slovakia, India, Japan and Thailand. For the most recent 10-year period, increasing trends were mainly observed in Central and South America, Asia and Central and Eastern Europe (Additional file 1: Figure S3). Larger significant increases were observed in Brazil (AAPC = 4.4%), the Republic of Korea (AAPC = 2.1%) and Japan (AAPC = 1.7%), whereas larger decreases were found in Austria (AAPC = − 3.7%), Israel (AAPC = − 3.2%) and the Czech Republic (AAPC = − 2.8%) (Additional file 1: Figure S3).

**Table 2:** Trends in ovarian cancer age-standardized rates for all ages

	Joinpoint analyses									
	Trend 1		Trend 2		Trend 3		Trend 4		AAPC	
	Years	APC	Years	APC	Years	APC	Years	APC	Last 10 years	
North America										
Canada^b^	1973–1995	0.3	1995–2000	−5.9^a^	2000–2007	0.2			1998–2007	−1.2
USA^b^	1973–1978	− 1.8	1978–1999	0.6^a^	1999–2002	−9.1^a^	2002–2014	− 1.4^a^	2005–2014	− 1.4^a^
US black^b^	1973–1994	0.5	1994–2014	−1.7^a^					2005–2014	− 1.7^a^
US white^b^	1973–1978	−1.8	1978–1999	0.8^a^	1999–2002	−9.3	2002–2014	−1.6^a^	2005–2014	−1.6^a^
Central and South America										
Brazil^b^	1988–2007	4.4^a^							1998–2007	4.4^a^
Colombia^b^	1983–2007	0.6							1998–2007	0.6
Costa Rica	1980–2007	0.5							1998–2007	0.5
Ecuador^b^	1985–2007	1.3							1998–2007	1.3
Western Europe										
Austria	1990–1997	0.8	1997–2009	−3.7^a^					2000–2009	−3.7^a^
France	1980–1987	0.1^a^	1987–1995	− 0.4^a^	1995–2003	− 0.8^a^	2003–2012	−1.2^a^	2003–2012	−1.2^a^
Germany^b^	1973–2007	0.2							1998–2007	0.2
The Netherlands	1989–2016	−1.5^a^							2007–2016	−1.5^a^
Switzerland^b^	1973–2007	−0.8^a^							1998–2007	−0.8^a^
Northern Europe										
Denmark	1973–2000	− 0.4^a^	2000–2014	− 2.4^a^					2005–2014	− 2.4^a^
Estonia	1973–2007	0.1							1998–2007	0.1
Finland	1973–1995	0.8^a^	1995–2014	−1.0^a^					2005–2014	−1.0^a^
Iceland	1973–2014	−1.7^a^							2005–2014	−1.7^a^
Ireland	1994–2013	−1.1^a^							2004–2013	−1.1^a^
Latvia	1988–2007	0.6^a^							1998–2007	0.6^a^
Lithuania	1978–2007	0.5^a^							1998–2007	0.5^a^
Norway	1973–1998	−0.0	1998–2014	−1.6^a^					2005–2014	− 1.6^a^
Sweden	1973–1987	− 0.8^a^	1987–2014	−2.1^a^					2005–2014	−2.1^a^
United Kingdom^b^	1975–1986	1.8^a^	1986–2007	0.0					1998–2007	0.0
Southern Europe										
Croatia	1988–2000	3.2^a^	2000–2014	−2.1^a^					2005–2014	− 2.1^a^
Italy^b^	1978–1981	7.1	1981–1986	−5.0	1986–1998	0.9	1998–2007	−2.0^a^	1998–2007	− 2.0^a^
Malta	1993–2009	−0.4							2000–2009	−0.4
Slovenia	1973–2013	−0.0							2004–2013	−0.0
Spain^b^	1973–1976	28.9^a^	1976–2007	1.2^a^					1998–2007	1.2^a^
Central and eastern Europe										
Bulgaria	1993–2007	1.7^a^							1998–2007	1.7^a^
Czech Republic	1977–2000	1.4^a^	2000–2014	−2.8^a^					2005–2014	−2.8^a^
Poland^b^	1978–2006	1.0^a^							1997–2006	1.0^a^
Russian Federation	1993–2008	0.8^a^	2008–2012	−0.2	2012–2015	1.9^a^			2006–2015	0.7
Slovakia	1973–1990	1.5^a^	1990–2010	0.3					2001–2010	0.3
Asia										
China^b^	1983–1993	−1.9^a^	1993–2000	3.0^a^	2000–2007	−0.8			1998–2007	− 0.0
India^b^	1978–2007	0.3							1998–2007	0.3
Israel	1973–1994	0.5	1994–2007	−3.2^a^					1998–2007	−3.2^a^
Japan	1975–1982	2.0^a^	1982–1985	9.7	1985–2012	1.7^a^			2003–2012	1.7^a^
Philippines^b^	1983–1988	−6.9^a^	1988–1995	8.3^a^	1995–1998	−8.2	1998–2007	1.8	1998–2007	1.8
Republic of Korea	1995–2005	−0.8	2005–2014	2.1^a^					2005–2014	2.1^a^
Singapore	1973–1975	−17.4	1975–1978	23.1	1978–2007	0.1			1998–2007	0.1
Thailand^b^	1983–2007	1.4^a^							1998–2007	1.4^a^
Oceania										
Australia	1982–1993	0.3	1993–1996	−3.1	1996–2013	−0.6^a^			2004–2013	−0.6^a^
New Zealand	1983–1994	0.4	1994–2013	−1.7^a^					2004–2013	−1.7^a^

*APC* annual percent change, *AAPC* average annual percent change

^a^The APC or AAPC is statistically different from zero

^b^Cases and population data of all registries were pooled to ensure the largest geographic coverage and obtain estimated a proxy of the national incidence

**Fig. 4:**
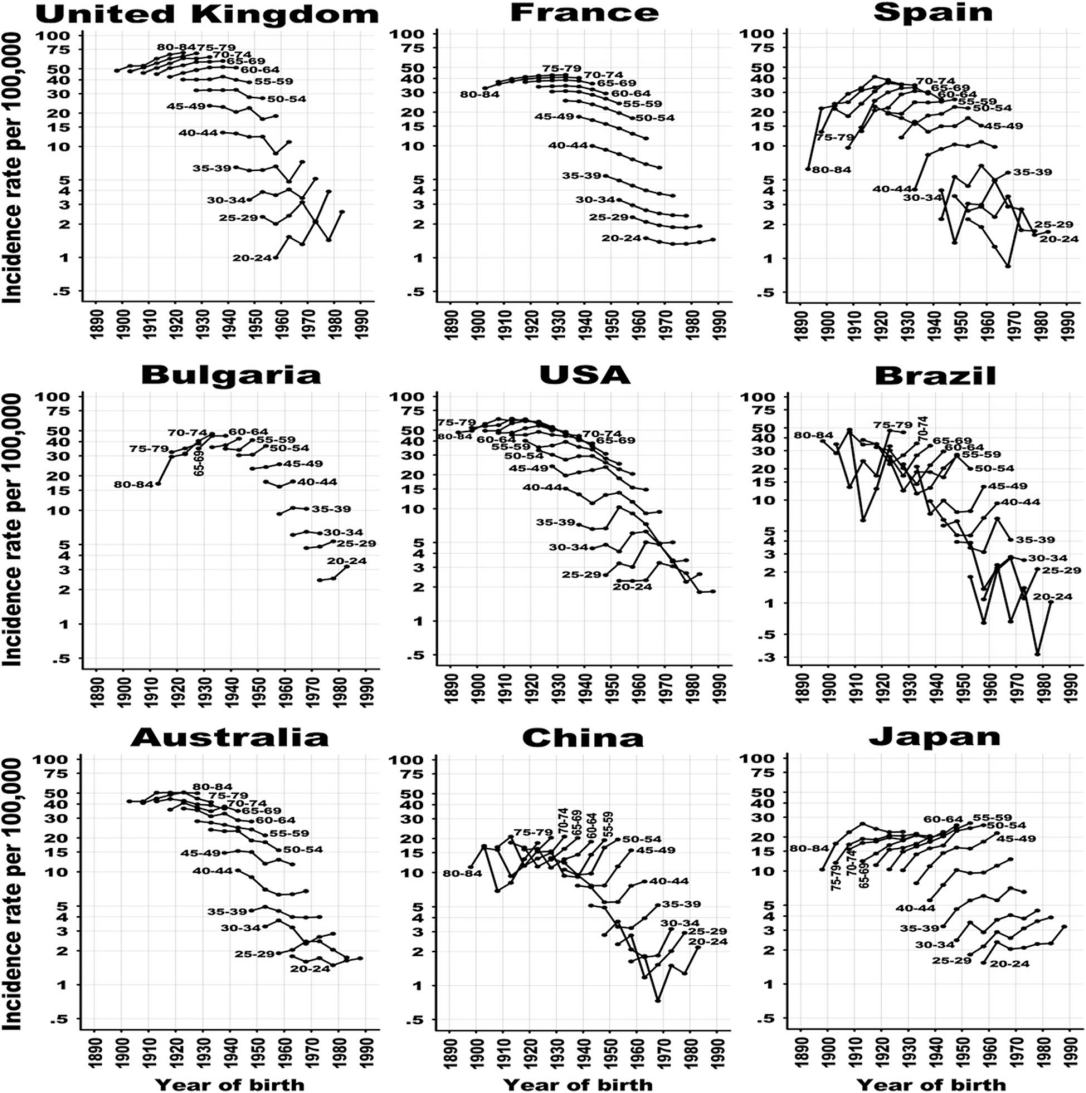
Ovarian cancer incidence rates per 100,000 women by year of birth for nine selected countries from each region. For each graph, the rates in 5-year age groups (e.g., 20–24, 25–29, …, 80–84) are plotted

Figure 4, Additional file 1: Figure S4 and S5, shows the 5-year age-specific OC incidence rates by birth cohort. The non-parallel appearance of the observed incidence rates versus the birth cohort across age groups indicates a strong cohort effect, as seen in almost all countries. For countries with age-specific differences, the incidence rate increased in the recent birth cohorts with age > 70 years in Denmark and Germany, age > 65 years in Finland, age > 50 years in Poland and Thailand, age > 40 years in Ecuador, age > 35 years in Latvia and age > 30 years in Korea. The other age groups of these countries show a decreasing trend. The incidence rate increased in the recent birth cohorts with age < 30 years in the Netherlands, Ireland and France, age < 35 years in Norway, age < 45 years in Russia, New Zealand and Singapore, age < 50 years in the UK, Slovenia and Estonia, and age < 70 years in Japan. The incidence rate decreased in the Netherlands between 30 and 65 years old, in France between 30 and 70 years old, and in the UK between 50 and 60 years old.

**Fig. 5:**
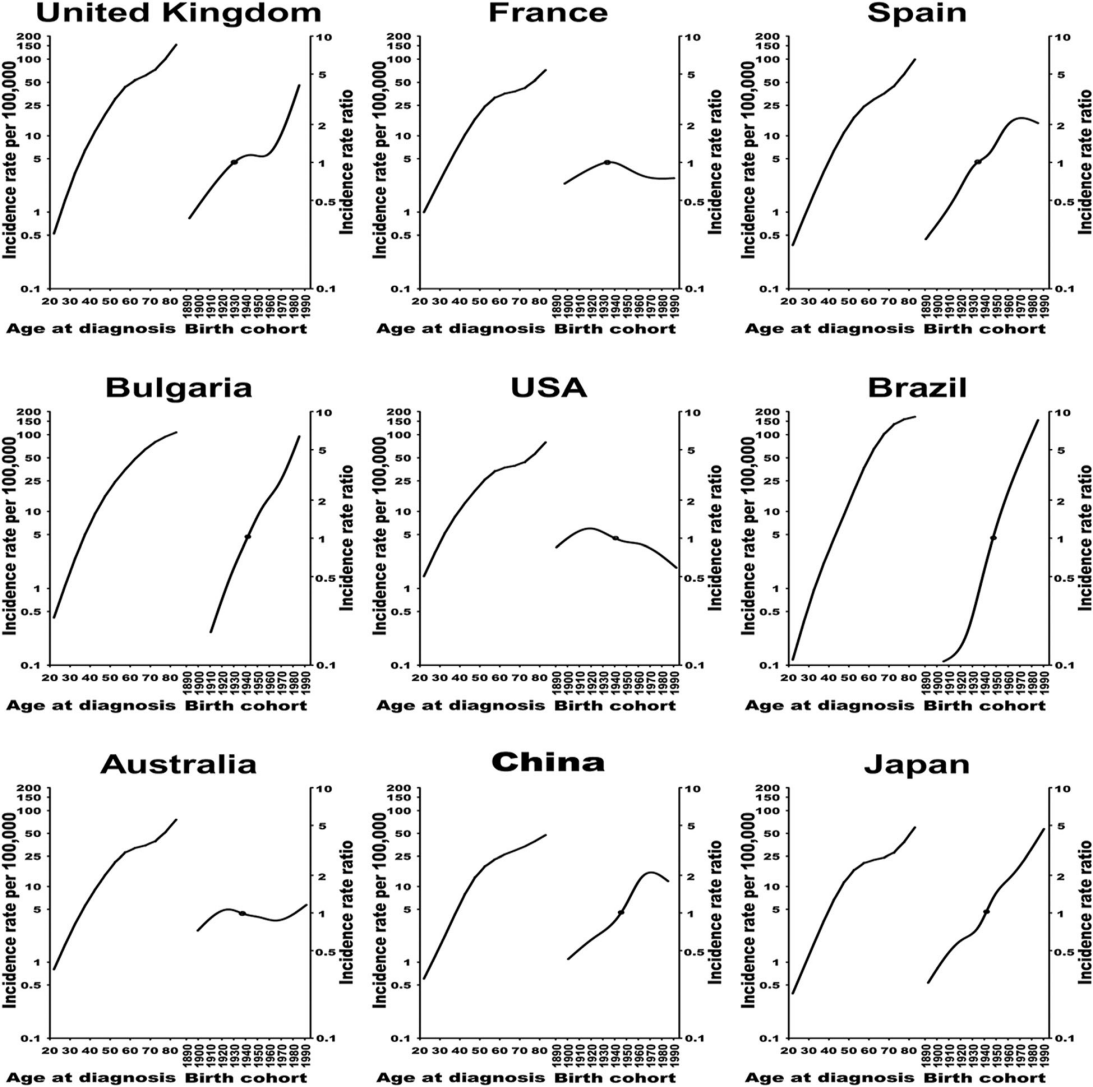
Fitted age-specific ovarian cancer incidence rates per 100,000 women (left) and incidence rate ratios by birth cohort (right) in nine selected countries from each region. The default is for the reference points at the median value (with respect to the number of cases) for the cohort to be variable

Figure 5, Additional file 1: Figure S6 and S7, depicts the graphs for the age and cohort effects. The incidence rates increased sharply with age in most countries. We observed four patterns of IRR for birth cohort effects. First, a continuous increase was found in Bulgaria, Estonia, Germany, Italy, Lithuania, Malta, Russia, Slovakia, Slovenia, the UK, Australia, Brazil, Costa Rica, Ecuador, India, Japan, Singapore and Thailand. Second, a trend in IRR analogous to a v-shaped curve was observed in Iceland, the Netherlands, New Zealand, Finland and the Philippines. The IRR in the Philippines decreased among birth cohorts from 1900 to 1920 and increased rapidly from 1920 onwards, while that in Iceland decreased slightly before the 1960–1970 birth cohort and increased from 1970 onwards, and that in the Netherlands, Finland and New Zealand increased after the 1970–1980 birth cohort. Third, a trend in IRR similar to an inverted v-shaped curve was observed in several countries. For example, the IRR of cohort effects in the USA and the USA White population increased among birth cohorts from 1890 to 1920 and decreased from 1920 onwards; that in France increased among birth cohorts from 1890 to 1930 and decreased from 1930 onwards; that in Norway and Switzerland increased among birth cohorts from 1890 to 1940 and decreased from 1940 onwards; that in Czech Republic and Ireland decreased from 1950 onwards; that in Croatia, Latvia, Poland, Canada, Israel and the USA Black populations decreased from 1960 onwards; and that in China, Spain, Colombia and the Republic of Korea decreased from 1970 onwards. Fourth, a consistent decrease in IRR was detected in Austria, Denmark and Sweden. For recent generations of the 41 countries, the IRR increased in Estonia, Finland, Iceland, Lithuania, the UK, Germany, the Netherlands, Italy, Malta, Slovenia, Bulgaria, Russia, Australia, New Zealand, Brazil, Costa Rica, Ecuador, India, Japan, the Philippines and Thailand.

## Discussion

In this study, we observed a large global variation in the ASR of OC. During the most recent 10-year period, increasing trends were mainly observed in Central and South America, Asia and Central and Eastern Europe. We also found visibly increasing IRR among recent birth cohorts in most countries worldwide.

The birth cohort effect indicates that individuals born in the same time period tend to adopt similar lifestyles that may influence their carcinogenic risks in specific ways. Changing lifestyle habits, including cigarette smoking, diet and oral contraceptive pills (OCPs), and obesity could have crucial impacts on the birth cohort risk of OC and hence influence national OC incidence trends.

There is a biological plausibility between smoking and OC; that is, researchers have discovered adducts of benzo(a) pyrene in the ovarian follicular cells of women who have ever smoked, and these adducts would increase DNA damage risk through a direct carcinogenic effect [18]. Women who have ever smoked had a 6% higher risk of OC than did those who have never smoked [19]. The global smoking prevalence among females (≥15 years old) declined from 10.6% in 1980 to 6.2% in 2012, with a decline of 1.7% every year [20]. From 1980 to 2012, smoking prevalence among females mainly declined in the Americas and Oceania countries, especially in North America, Australia, and New Zealand, while prevalence started to decline moderately during recent years in European countries and has changed little in Asian countries [20]. For example, the smoking prevalence in Canada, the USA, Denmark, Iceland, Norway, Sweden, the United Kingdom and Israel was higher than 20% in 1980 and declined by greater than 2% annually, whereas the prevalence in Bulgaria, which was similar to that of the above-mentioned countries in 1980, increased with statistical significance since 1980 [20]. The trends in smoking prevalence could partly explain OC trends in these regions and countries for the study period, especially for the most recent 10 years. In 2012, the greatest smoking prevalence among females occurred in European regions, followed by Oceania and North America, while smoking prevalence in Asian and African countries was low; that is, smoking prevalence across high-income countries in 2012 varied greatly, from less than 15% in Canada, Iceland, Israel, Japan, Sweden, and the USA to greater than 26% in Bulgaria and France, while in many middle-income countries, the smoking prevalence never exceeded 5% [20]. The global smoking prevalence pattern in 2012 also seems to explain the global patterns in OC incidence in 2012 from our study that the highest ASR for OC was observed in Central and Eastern Europe, Northern Europe, Southern Europe, North America, and Oceania. Generation-specific smoking prevalence data can also help explain the trends of OC better. In Japan, smoking prevalence continuously increased in the 1930s–1970s birth cohort among women [21]. In Germany, the smoking prevalence increased from 20% in the 1926–1930 birth cohort to approximately 50% in the 1966–1970 birth cohort among women [22]. This finding seems to be consistent with the increasing birth cohort risk of OC in these countries in our study.

Mechanistically, red meat and processed meat are sources of iron, high salt content, saturated fats, and several mutagens, including N-nitroso and nitrosamine compounds, heterocyclic amines and polycyclic aromatic hydrocarbons, which are associated with DNA damage and an increased risk of OC [23]. A healthy dietary pattern was associated with a reduced 14% risk of OC, and a western-style dietary pattern, such as high intakes of red meat and processed meat, was associated with an 19% increased risk of OC [24]. In 2010, the average red meat intake met the recommended standards of ≤1 (100 g) serving/week in only 5 of 187 countries (representing 20.3% of the world’s population). Global average red meat consumption increased by 1.5 g/day from 1990 to 2010, while the consumption significantly increased by 8.3 g/day in East Asia [25]. The greatest increases in intake occurred in Latvia (+ 15.2 g/day) and the Republic of Korea (+ 13.4 g/day), while a large decline was observed in Canada (− 7.1 g/ day), the Netherlands (− 6.1 g/day) and the USA (− 4.7 g/day) [25]. The average processed meat intake met the recommended standards of ≤1 (50 g) serving/week in 55 of 187 countries (representing 38.5% of the world’s population) in 2010 [25]. Some countries consume more red meat and processed meat (e.g., American nations, such as Colombia; and European nations, such as Poland, Russia, Latvia and Lithuania) [25]. The change in red meat intake and the dietary pattern worldwide also seems to partly explain the pattern and trends in OC incidence in the regions and countries examined in our study. Notably, the birth cohort effect implies the importance of diet in early life. Diet may explain part of the increased OC risk of recent birth cohorts in Japan, since Japanese dietary patterns have shifted greatly to Western-style meals over recent decades [26]. The increasing risk for the cohort born after the 1920s in Korea from our study might be partly explained by the gradual westernization of lifestyles and dietary pattern towards increased meat and fat consumption in Korea [27].

Compared with normal weight women, obese women would reduce serum progesterone levels because of an increase in anovulatory cycles, while progesterone has a protective impact on ovarian carcinogenesis [28]. In addition, obesity increased insulin and insulin–like growth factor–1 levels, which would increase OC risk [29]. Hence, overweight women had a 7% higher risk of OC, and obese women had a 28% higher risk [30]. A population-based study indicated that the estimated population attributable fraction of OC cases in 2012 associated with excess body mass index (defined as 25 kg/m (2) or greater) is 33% for Eastern Europe, 30% for Northern Europe, 30% for Southern Europe and 34% for North America [31]. These findings may partly explain the highest ASR of OC in Europe and North America in 2012. The proportion of adults with body mass index ≥25 kg/m^2^ increased from 29.8 to 38.0% for females from 1980 to 2013 worldwide [32]. The proportion of women with a high body mass index increased even faster than the global average in the high-income countries included in our study (except Japan) [32]. The increasing prevalence of obesity due to lifestyle changes is likely to cause a birth cohort effect. In Australia, a quasi–V-shaped obesity trend was observed for females for birth cohorts from 1915 to 1980 [33], which seems to be consistent with the changes in the birth cohort risk of OC in our study. The prevalence of obesity among women increased from 5.0 to 10.1% from 1993 to 2009 in China [34] and from 8.0 to 16.5% from 1987 to 2012 in Spain [35]. Such an increase in obesity may explain in part the increasing birth cohort risk of OC in these countries.

OCPs are a well-established protective factor for OC. The biological mechanisms underlying this association include that OCPs could suppress ovulation, lower follicle-stimulating hormone, eliminate the midcycle surge of luteinizing hormone and reduce stromal cell reactivity, all of which would reduce the risk of OC [36]. The risk of OC decreased by 20% for each 5 years of OCP use [37]. Furthermore, the reduction in OC risk persisted for more than 30 years after OCP use had ceased [38]. In Northern and Western Europe and North America, where the use of OCPs was earlier and more widespread, the favourable trends in OC can partly be attributed to its long-term protection. OCPs were introduced in Europe in the early 1960s [39]. The estimated proportion of women aged 15–45 taking OCPs was 20 to 30% in the UK, Demark and Sweden in the mid-1970s and approximately 30 to 40% of women aged 15–45 in Northern Europe by the late 1980s and early 1990s [40–42]. In France, the proportion of women aged 20–44 who regularly take OCPs increased from 28.3% in 1978 to 45.4% in 2000 [43]. By 2010, 79% of women aged 15–29 were taking OCPs in France [43]. As a consequence, the differences in the introduction time of OCPs and the prevalence of OC may partly explain why birth cohorts after the 1920s–1940s in most countries of Northern and Western Europe and North America showed a decreasing trend in birth cohort risk. Our study indicated that the IRR of OC increased until the cohort born approximately 1918 in the USA and 1923 in Australia, and these individuals were the first generation to use OCPs [44]. In France, the change in birth cohort risk in our study can be explained by the reduction in the cumulative risk in the cohorts born from 1930 onwards, corresponding to the advent of OCPs among the female population [43]. Furthermore, in Bulgaria of Central and Eastern Europe, the continuous and rapidly increasing OC birth cohort trends may be explained in part by the low oral contraceptive use (6.2% in 2007) [42]. In Asia, oral contraceptive use was also very low; that is, the prevalence of OCP use in India, China, and the Republic of Korea was less than 4% before 2010 [42], which might explain in part the continuous increase in OC rate in most Asian countries due to the low prevalence of OCP use. In Japan, oral contraceptives were released for general use only in 1999 [45], and the prevalence of OCP among women aged 15–49 years was only 1.1% in 2015 [46], which may also explain in part the increasing trends and birth cohort risk in OC incidence in Japan. The prevalence of OCP use in Central and Eastern Europe and Asian countries was markedly lower than those in western countries, such as the USA (16.0%) and France (39.5%) [46]. Thus, the decreasing trends and birth cohort risk of OC incidence are large in most countries of Northern and Western Europe, North America and Oceania, while the increasing trends and birth cohort risk of OC incidence occurred in Central and Eastern Europe and Asian countries.

Pregnancy could reduce the lifetime number of ovulatory cycles [47], lower gonadotropin secretion and subsequent oestrogen stimulation of the ovarian surface epithelium [48], and clear precancerous cells from the ovary [49], all of which would reduce the risk of OC. The level of protection increases with the number of childbirths (relative risk per child, 0.90), and compared to nulliparous women, the risk of OC among parous women decreased 30% [50]. Reproductive factors also seem to be important in influencing OC trends worldwide. The total fertility rates per woman in Western and Northern European countries, such as France, Denmark, Iceland, Ireland and Norway, were 2.0 and have slightly increased in recent decades [51]. The fertility rates in North American countries also slightly increased from 1.7 in 1975 to 1.9 in 2015, while the total fertility rates in Oceanian countries slightly decreased from 2.7 to 2.4 [52]. In addition, downward trends in OC incidence from the 1970s to the 1990s in western countries could be partly explained by the increasing fertility rates after World War II, since females of the baby boom generation reached child-bearing ages [44]. However, with the influence of family planning and western culture, the parity has substantially decreased in most Asian countries, some Southern American countries and Central and Eastern European countries since 1965 (from an average of 6 to < 3 by 2000) [53]. Indeed, over the last 4 decades (1975–2015) in Asia, the total fertility rates per women decreased from 5.0 to 2.3 in India, from 5.5 to 2.9 in the Philippines and from 3.9 to 1.5 in Thailand [52]. In China, the mean parity decreased from 4.9 to 1.1 for urban women and from 5.9 to 1.4 for rural women born between 1930 and 1974 [54]. In South America, the total fertility rates per woman decreased from 4.3 to 1.7 in Brazil and from 5.1 to 2.4 in Ecuador from 1975 to 2015 [52]. In Central and Eastern Europe, the total fertility rates per woman in Bulgaria, Poland and Russia decreased from 2.5 in 1975 to 1.3 in 2015 [52]. The parity of fertile women between 15 and 49 years old in these regions and countries decreased by almost half during the half century, which may partly explain why the successive birth cohort effect from 1900 to 1935 onwards in these countries shows an upward trend, especially for Asian and Southern American countries with larger decreases in fertility rates.

Oestrogen stimulation of ovarian tissue via various pathways after hormone replacement therapy (HRT) use may support the positive biological association between HRT and OC [55]. Compared with never-use HRT women, HRT users had a 37% higher risk of OC [56]. In the USA and the UK, the prevalence of HRT substantially increased before 2002 and then halved abruptly in 2002 following Women’s Health Initiative reports of increased risks of OC or other diseases [56, 57]. The patterns of HRT in Western and Northern Europe and Australia are similar to those observed in the USA and the UK [56]. Our results were consistent with these findings, suggesting an accelerated decline in OC incidence rates for middle- and older-aged women in these areas and countries after 2002. We speculated that the decreasing trends in OC may be due to the large reduction in the use of HRT after 2002.

Notably, the incidence trends of OC may also be partially influenced by changes in diagnostic facilities and disease classifications, particularly in high-income countries. Some improvement in the diagnosis and certification of OC in western countries has taken place from the 1980s to the 1990s since the echography, CT scan and endoscopy were introduced [39], thus leading to partial increasing incidence trends in OC in those countries during that period. In addition, in Central and Eastern European countries, the delayed introduction of advanced diagnosis and management method of OC [58] may have caused the recent increasing incidence trends of OC in these countries from our study. In India, new diagnostic equipment was introduced, and diagnostic methods were concentrated in some large-scale hospitals from the mid-1980s to mid-1990s [59], so these changes may have instant effects on OC rates and led to the observed increasing incidence trends of OC during that period in our study. The declining trends in OC incidence after 2000 may be partly due to changes in the disease classification. Compared to ICD-O 2nd edition published by the World Health Organization in 1990, ICD-O 3rd edition, published in 2000 no longer considers ovarian neoplasms with borderline malignancy or low malignant potential as ovarian malignant tumours, while borderline tumours accounted for approximately 15% of all OC cases [60]. For example, a recent study indicated that the slight decreasing trends in OC incidence in Denmark after 2000 may be attributed to diagnostic changes in pathological standards for ovarian carcinomas versus ovarian borderline tumours and the different assessments among pathologists [61]. In addition, diversities in cancer screening practices could influence comparisons of OC rates between countries in our study. However, our study could not evaluate how these factors exactly influence the trends in OC incidence, which is one of the major limitations in our study and should be explored further with studies in the future.

Ecological fallacy is another major limitation in our study. The included databases in our study, such as the GLOBOCAN 2012, CI5 and SEER databases, were derived from different cancer registries at different ecological levels, and these regional registry data were aggregated to obtain an estimate of national incidence. In our study, ecological fallacy occurs when these regional registry data are aggregated, and conclusions drawn on the basis of a group-level analyses differ from those that would have been drawn based on individual-level data. Thus, ecological fallacy must be considered as a possible interpretation of the findings in our study, and these findings must be interpreted with caution when conclusions drawn on country-level were inferred towards an individual level from our study, especially for the above-mentioned factors.

There also exists other limitations in our study. First, in developing countries, regular medical records could be incomplete, and population estimates could be imprecise, which could affect the accuracy of OC incidence rates. In developing countries, cancer registries are usually established in metropolises, and the prevalence of unhealthy lifestyle behaviours is higher than that in the general population. Second, there may be an underestimate of the regional cancer registries with limited resources. However, there may be an overestimate of the high-quality regional cancer registries with long-term OC prevention strategies. Third, random variation in statistical analysis could occur in low-risk populations on account of small numbers. Finally, our study did not include African countries because of a lack of historical data in CI5. As a result, the findings of our study should be interpreted with caution.

Despite the above-mentioned limitations, this study systematically assessed geographical variations and long-term trends in OC incidence worldwide, focusing on the differences between countries and birth cohort effects. The brilliant highlights are that we detected the geographical disparities in regions and 41 countries worldwide and explored birth cohort trends, which is a more systematic analysis than that in previous studies. In addition, we adopted the CI5 database with rigorous data-quality standards.

## Conclusions

The major finding of our study is the increasing IRR of OC observed by birth cohort for most countries in Asia, Central and Eastern Europe, and Central and South America. The increasing trends may be due to the increased prevalence of smoking, the westernized dietary patterns, obesity, and the decreased prevalence of parity, while the decreasing trends may be due to the increased prevalence of OCPs and the decreased prevalence of HRT. Public health and cancer prevention specialists should pay more attention to countries with increasing risk of OC. The reason for the increasing OC risk for recent birth cohorts in these countries should be investigated with further epidemiology studies.

## Supplementary information

**Additional file 1:** **Fig. S1** Temporal trends in age-standardized (world 1960 Segi population) ovarian cancer incidence rates per 100,000 women for European countries for all ages from 1973 to 2015. **Fig. S2** Temporal trends in age-standardized (world 1960 Segi population) ovarian cancer incidence rates per 100,000 women for non-European countries for all ages from 1973 to 2015. **Fig. S3** Average annual percentage change in ovarian cancer incidence rates for all ages in the most recent 10-year period available. * Average annual percent change is significantly different from zero. **Fig. S4** Ovarian cancer incidence rates per 100,000 women by year of birth for European countries. For each graph, the rates in 5-year age groups (e.g., 20–24, 25–29, …, 80–84) are plotted. **Fig. S5** Ovarian cancer incidence rates per 100,000 women by year of birth for non-European countries. For each graph, the rates in 5-year age groups (e.g., 20–24, 25–29, …, 80–84) are plotted. **Fig. S6** Fitted age-specific ovarian cancer incidence rates per 100,000 women (left) and incidence rate ratios by birth cohort (right) in European countries. The default is for the reference points at the median value (with respect to the number of cases) for the cohort to be variable. **Fig. S7** Fitted age-specific ovarian cancer incidence rates per 100,000 women (left) and incidence rate ratios by birth cohort (right) in non-European countries. The default is for the reference points at the median value (with respect to the number of cases) for the cohort to be variable.

## Data Availability

National OC incidence estimates in 2012 for 184 countries were extracted from the GLOBOCAN database (http://globocan.iarc.fr). For the 41 countries studied, national incidence data were available for 26 countries, and registry incidence data were available for 15 countries. National incidence data sources: Austria (http://eco.iarc.fr/EUREG/AnalysisT.aspx), Croatia (https://www.hzjz.hr/sluzba-epidemiologija-prevencija-nezaraznih-bolesti/publikacije-odjel-za-maligne-bolesti/), Czech Republic (http://www.svod.cz/?sec=aktuality&lang=en#), Denmark (http://www-dep.iarc.fr/NORDCAN/english/frame.asp), Finland (http://www-dep.iarc.fr/NORDCAN/english/frame.asp), France (http://invs.santepubliquefrance.fr/Dossiers-thematiques/Maladies-chroniques-et-traumatismes/Cancers/Surveillance-epidemiologique-des-cancers/Estimations-de-l-incidence-de-la-mortalite-et-de-la-survie/Estimation-de-l-incidence-et-de-la-mortalite-par-cancer-en-France-metropolitaine-entre-1980-et-2012-Tumeurs-solides), Iceland (http://www-dep.iarc.fr/NORDCAN/english/frame.asp), Ireland (http://www.ncri.ie/data/incidence-statistics), Malta (http://eco.iarc.fr/EUREG/AnalysisT.aspx), the Netherlands (http://www.cijfersoverkanker.nl/selecties/dataset_1/img594e1bb7eb0e9), Norway (http://www-dep.iarc.fr/NORDCAN/english/frame.asp), Russia (http://www.oncology.ru/service/statistics/malignant_tumors/), Slovakia (http://www.nczisk.sk/Publikacie/Pages/Edicia-analytickych-publikacii.aspx), Slovenia (http://www.onko-i.si/dejavnosti/epidemiologija_in_register_raka/registri_raka/), Sweden (http://www-dep.iarc.fr/NORDCAN/english/frame.asp), Australia (http://www.aihw.gov.au/acim-books/), New Zealand (http://www.health.govt.nz/nz-health-statistics/health-statistics-and-data-sets/cancer-data-and-stats), Japan (http://ganjoho.jp/en/professional/statistics/table_download.html), the Republic of Korea (http://ncc.re.kr/cancerStatsList.ncc?searchKey=total&searchValue=&pageNum=1), Bulgaria (http://ci5.iarc.fr/Default.aspx), Costa Rica (http://ci5.iarc.fr/Default.aspx), Estonia (http://ci5.iarc.fr/Default.aspx), Israel (http://ci5.iarc.fr/Default.aspx), Latvia (http://ci5.iarc.fr/Default.aspx), Singapore (http://ci5.iarc.fr/Default.aspx). Registry incidence data sources: Canada, Brazil, Colombia, Ecuador, Germany, Switzerland, the United Kingdom, Italy, Spain, Poland, China, India, the Philippines, Thailand (data of these countries were drawn from the Cancer Incidence in Five Continents database: http://ci5.iarc.fr/Default.aspx), and the United States (data were drawn from the Surveillance, Epidemiology and End Results Program: www.seer.cancer.gov).

## Abbreviations

AAPC: Average annual percent change
ASR: Age-standardized rate
CI5: Cancer Incidence in Five Continents
HRT: Hormone replacement therapy
ICD-O: International Classification of Disease for Oncology
IRR: Incidence rate ratio
LOWESS: Locally weighted scatterplot smoothing
OC: Ovarian cancer
OCPs: Oral contraceptive pills
SEER: Surveillance, Epidemiology and End Results
UK: United Kingdom
USA: United States
