# Supplementary material for: Global patterns and trends in ovarian cancer incidence: age, period and birth cohort analysis
Source: BMC Cancer. 2019 Oct 22;19:984. doi: 10.1186/s12885-019-6139-6 (PMC6806513; doi:10.1186/s12885-019-6139-6)
Supplement: Supplementary file 1 — Additional file 1: Fig. S1 Temporal trends in age-standardized (world 1960 Segi population) ovarian cancer incidence rates per 100,000 women for European countries for all ages from 1973 to 2015. Fig. S2 Temporal trends in age-standardized (world 1960 Segi population) ovarian cancer incidence rates per 100,000 women for non-European countries for all ages from 1973 to 2015. Fig. S3 Average annual percentage change in ovarian cancer incidence rates for all ages in the most recent 10-year period available. * Average annual percent change is significantly different from zero. Fig. S4 Ovarian cancer incidence rates per 100,000 women by year of birth for European countries. For each graph, the rates in 5-year age groups (e.g., 20–24, 25–29, …, 80–84) are plotted. Fig. S5 Ovarian cancer incidence rates per 100,000 women by year of birth for non-European countries. For each graph, the rates in 5-year age groups (e.g., 20–24, 25–29, …, 80–84) are plotted. Fig. S6 Fitted age-specific ovarian cancer incidence rates per 100,000 women (left) and incidence rate ratios by birth cohort (right) in European countries. The default is for the reference points at the median value (with respect to the number of cases) for the cohort to be variable. Fig. S7 Fitted age-specific ovarian cancer incidence rates per 100,000 women (left) and incidence rate ratios by birth cohort (right) in non-European countries. The default is for the reference points at the median value (with respect to the number of cases) for the cohort to be variable. [file 12885_2019_6139_MOESM1_ESM.docx]

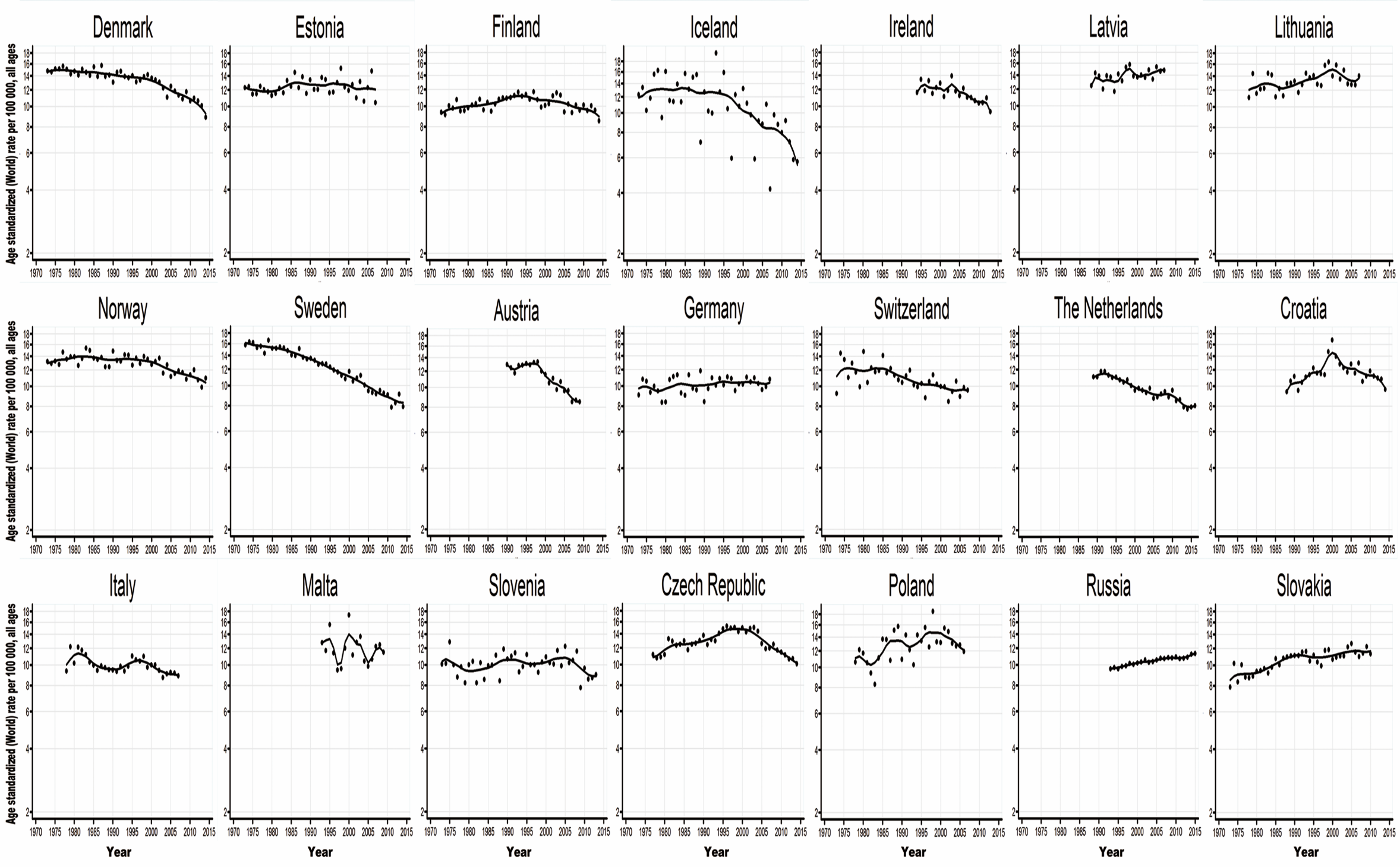


Figure S1. Temporal trends in age-standardized (world 1960 Segi population) ovarian cancer incidence rates per 100,000 women for European countries for all ages from 1973 to 2015.


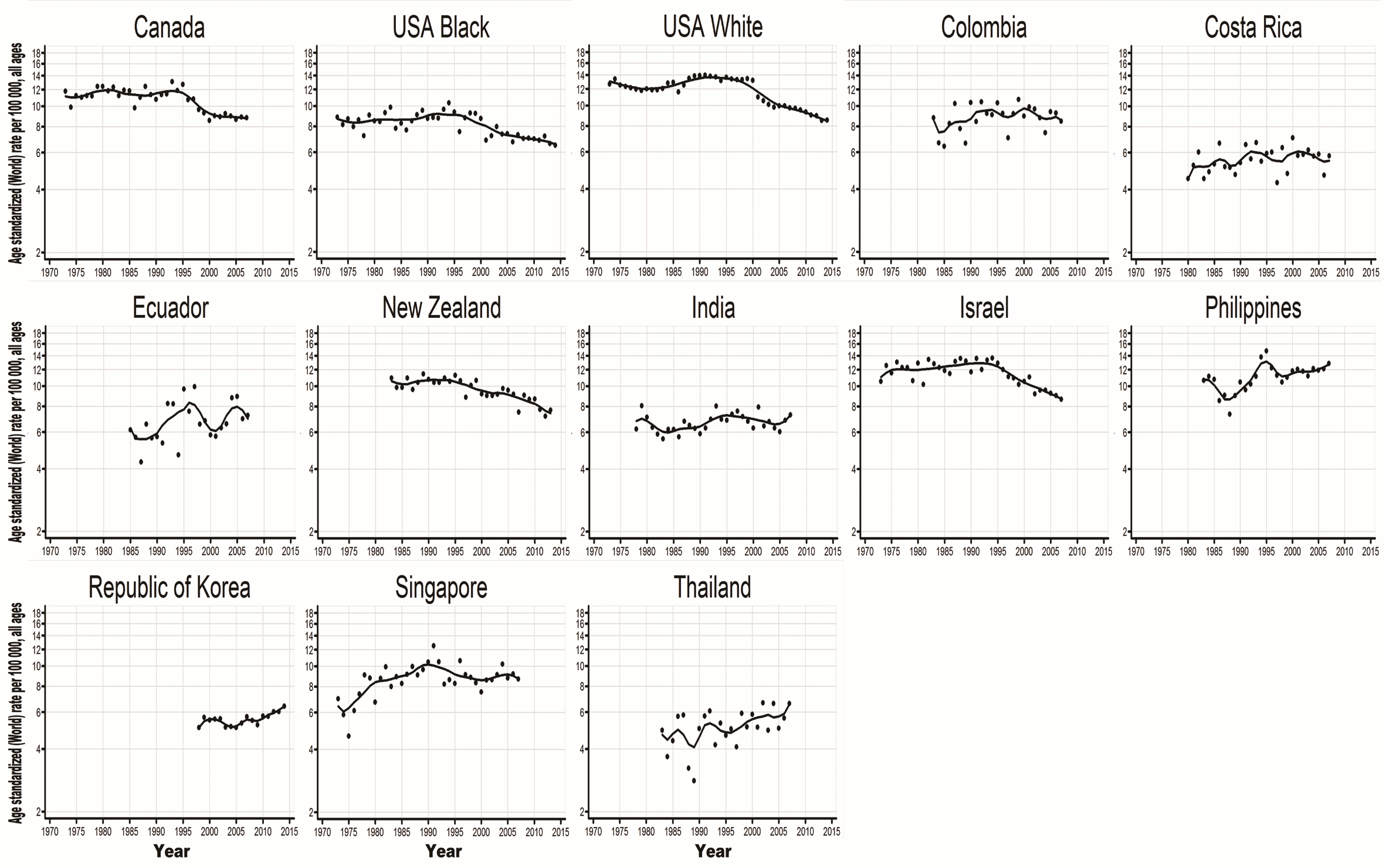


Figure S2. Temporal trends in age-standardized (world 1960 Segi population) ovarian cancer incidence rates per 100,000 women for non-European countries for all ages from 1973 to 2015.


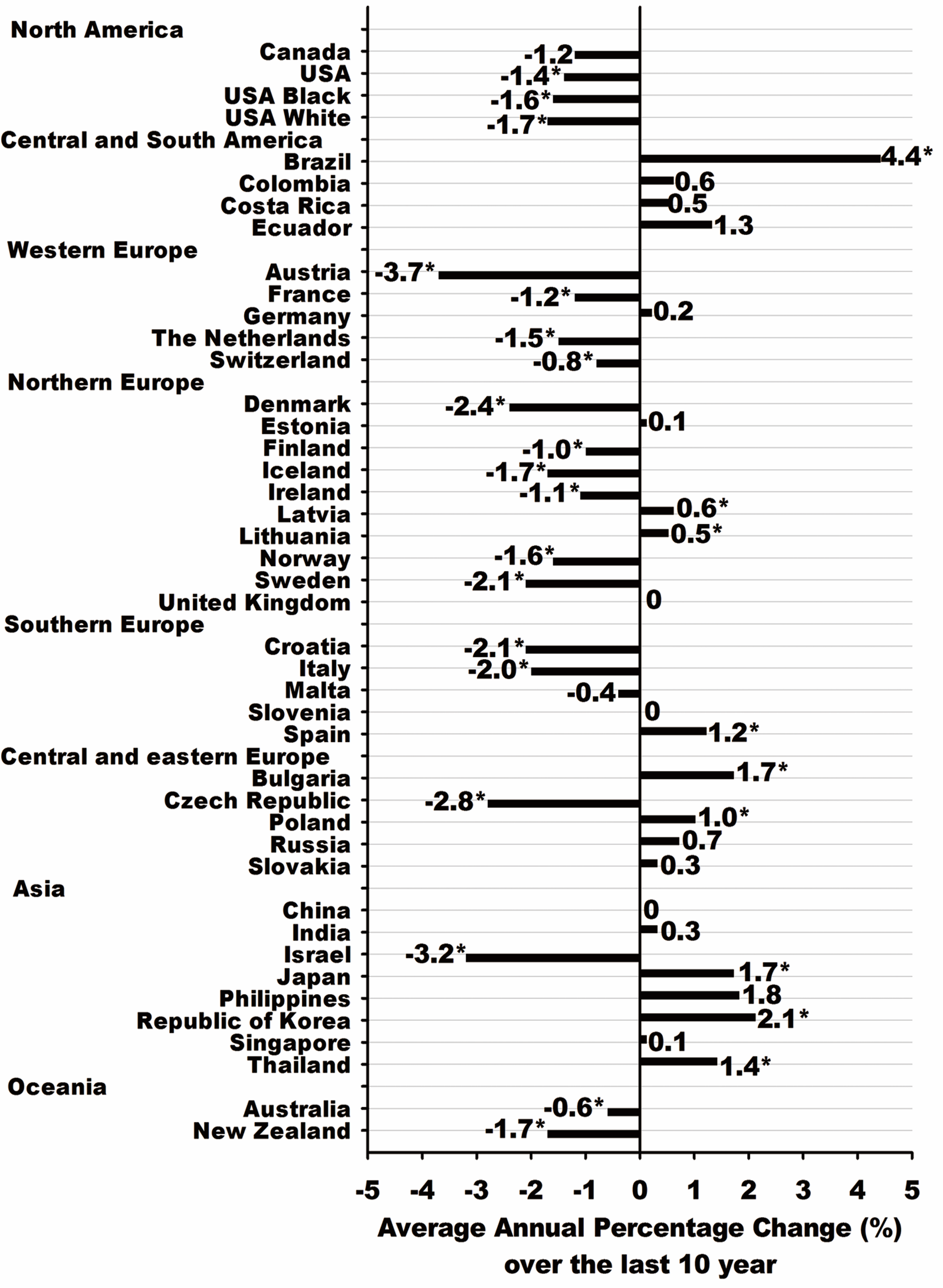


Figure S3. Average annual percentage change in ovarian cancer incidence rates for all ages in the most recent 10-year period available. * Average annual percent change is significantly different from zero.


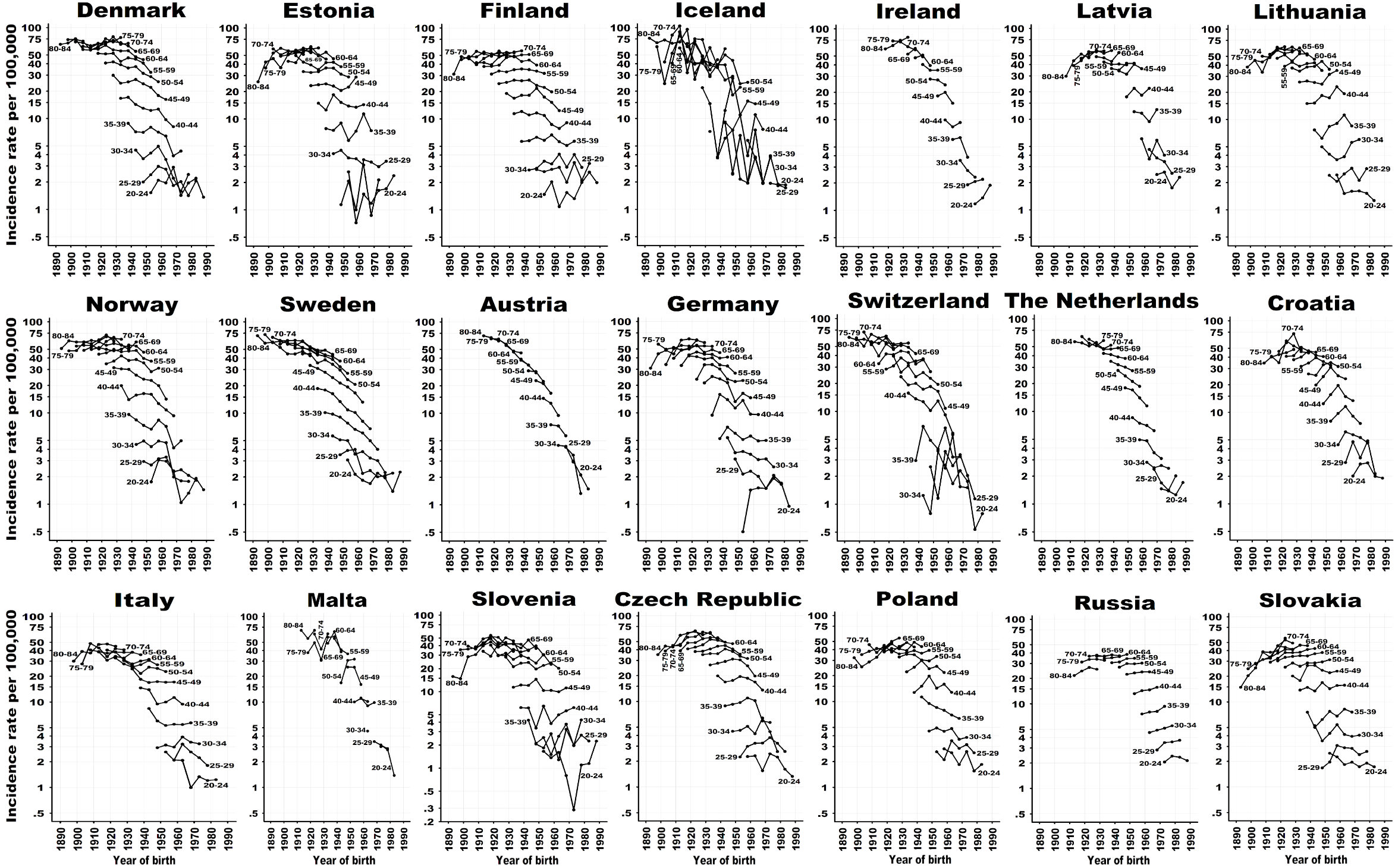


Figure S4. Ovarian cancer incidence rates per 100,000 women by year of birth for European countries. For each graph, the rates in 5-year age groups (e.g., 20–24, 25–29, …, 80–84) are plotted.


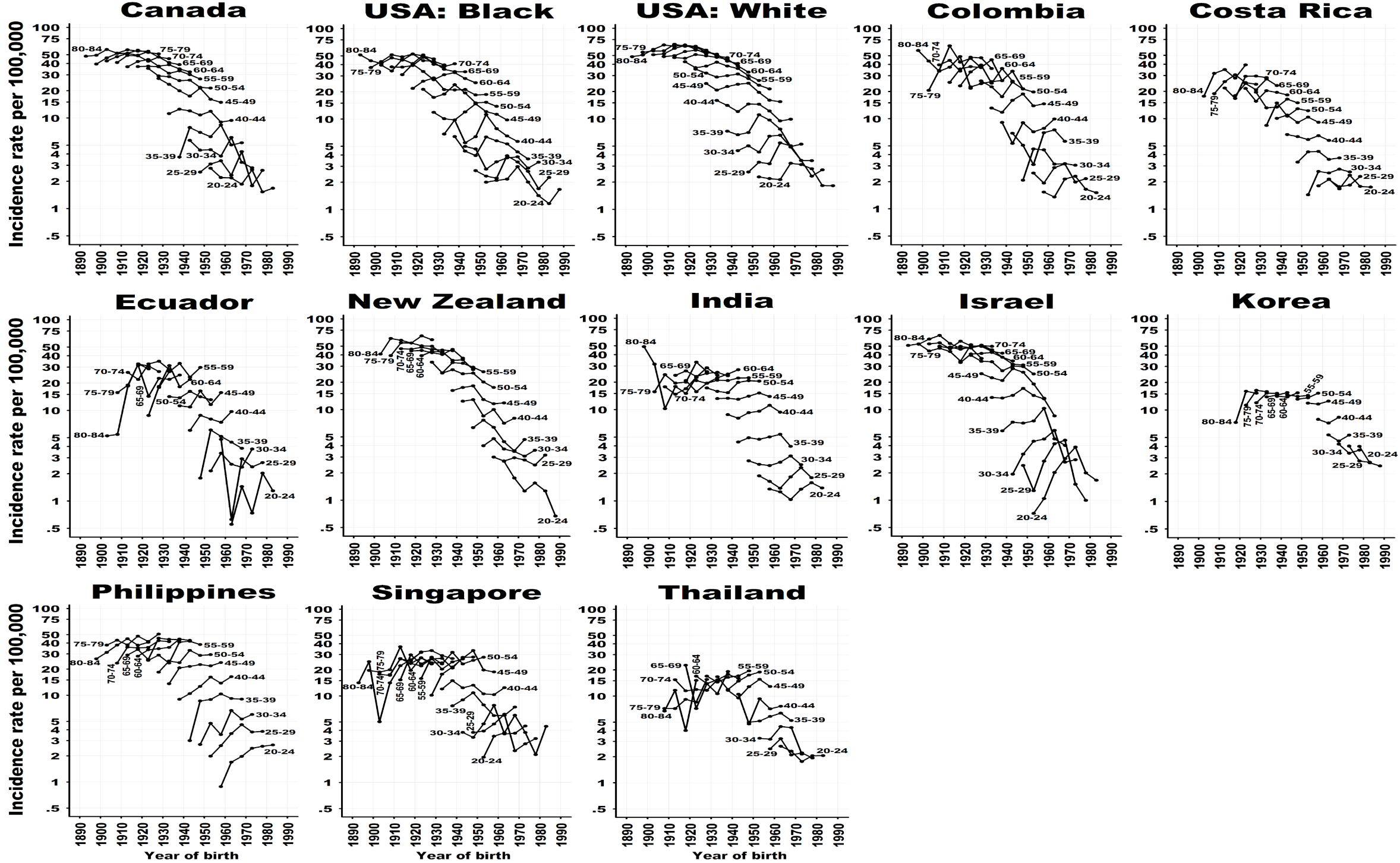


Figure S5. Ovarian cancer incidence rates per 100,000 women by year of birth for non-European countries. For each graph, the rates in 5-year age groups (e.g., 20–24, 25–29, …, 80–84) are plotted.





Figure S6. Fitted age-specific ovarian cancer incidence rates per 100,000 women (left) and incidence rate ratios by birth cohort (right) in European countries. The default is for the reference points at the median value (with respect to the number of cases) for the cohort to be variable.


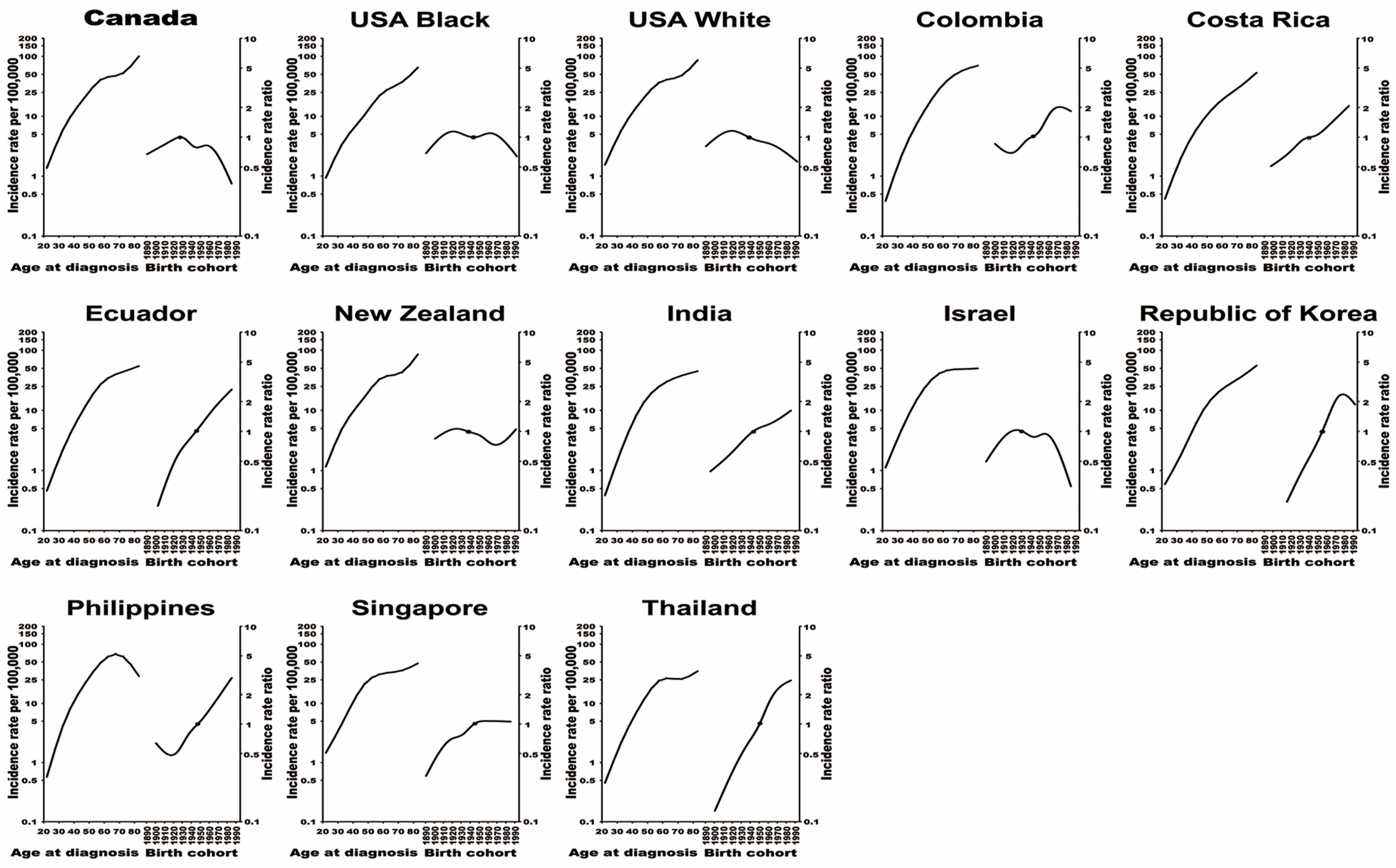


Figure S7. Fitted age-specific ovarian cancer incidence rates per 100,000 women (left) and incidence rate ratios by birth cohort (right) in non-European countries. The default is for the reference points at the median value (with respect to the number of cases) for the cohort to be variable.
